# The intersection between immunotherapy and laser interstitial thermal therapy: a multipronged future of neuro-oncology

**DOI:** 10.1080/02656736.2020.1746413

**Published:** 2020-07

**Authors:** Ethan S. Srinivasan, Eric W. Sankey, Matthew M. Grabowski, Pakawat Chongsathidkiet, Peter E. Fecci

**Affiliations:** a Department of Neurosurgery, Duke University School of Medicine, Durham, NC, USA; b Department of Neurosurgery, Duke University Medical Center, Durham, NC, USA; c Department of Neurosurgery, Cleveland Clinic, Cleveland, OH, USA

**Keywords:** Laser interstitial thermal therapy (LITT), hyperthermia, immunotherapy, cancer, neurosurgery, neuro-oncology

## Abstract

The rise of immunotherapy (IT) in oncological treatment has greatly improved outcomes in a number of disease states. However, its use in tumors of the central nervous system (CNS) remains limited for multiple reasons related to the unique immunologic tumor microenvironment. As such, it is valuable to consider the intersection of IT with additional treatment methods that may improve access to the CNS and effectiveness of existing IT modalities. One such combination is the pairing of IT with localized hyperthermia (HT) generated through technologies such as laser interstitial thermal therapy (LITT). The wide-ranging immunomodulatory effects of localized and whole-body HT have been investigated for some time. Hyperthermia has demonstrated immunostimulatory effects at the level of tumor cells, immune cells, and the broader environment governing potential immune surveillance. A thorough understanding of these effects as well as the current and upcoming investigations of such in combination with IT is important in considering the future directions of neuro-oncology.

## Introduction

While the emergence of effective immunotherapies has redrawn the landscape of modern oncology across various disciplines, their efficacy in the treatment of central nervous system (CNS) tumors such as glioblastoma (GBM) is limited. As such, a diverse range of synergistic treatment pathways has been employed to drive and sustain an adaptive antitumor response given the challenges presented by the unique immunologic microenvironment of the CNS. One such treatment paradigm involves the integration of two innovative modalities, laser interstitial thermal therapy (LITT) and immunotherapy (IT). This review seeks to examine the immunomodulating effects of LITT (and other thermal ablative schemas), and the rationale and future potential of concomitant IT.

Laser interstitial thermal therapy is a method of minimally invasive surgery first developed in the 1990s for deep-seated intracranial tumors. Since its inception, the use of LITT has expanded to a number of pathologies [1–6]. The procedure involves the stereotactic placement of a laser probe tip within an identified target lesion that then generates a focused distribution of thermal energy to produce coagulative necrosis for lesion destruction [7–11]. There are two commercial systems currently available for LITT, the NeuroBlate™ system from Monteris and the Visualase™ system from Medtronic, with some variation in their treatment protocols and construction. However, the overall technique and underlying mechanism are consistent across the marketplace [12–14]. The probe is placed using preprocedural magnetic resonance imaging (MRI) trajectory planning. Once it is in position, light energy travels through a fiberoptic cable to the probe tip centered within the region of interest [2,4–6,8,10,11,15–20]. MRI software is then used to generate maps of thermal change and tumor necrosis using the Arrhenius thermal dose model to guide administration of the therapy over the targeted area [21–24].

### LITT utilization and mechanism of action

Over the past 30 years, studies have documented the use of LITT for a range of neurosurgical pathologies, including primary brain tumors, recurrent metastases, radiation necrosis, epidural spinal metastases, and epilepsy [8,9,11,25–49]. Though large-scale randomized trials comparing LITT to more conventional methods of treatment are currently lacking, several smaller studies have demonstrated successful outcomes in otherwise non-surgical candidate patients for a number of common LITT applications. Most of the existing LITT studies have focused on primary brain tumors, given their extremely grim prognosis after exhaustion of conventional therapies [8,11,25–28,31]. With current standard ofcare, GBM carries a dismal median survival of just under 21 months [50–56].

Banerjee et al. reviewed the literature for LITT in neuro-oncology and found improved to comparable median overall survival of 20.9 months from diagnosis of recurrence in grade III/IV malignant gliomas relative to conventional treatments of chemotherapy, open surgery, high-dose brachytherapy, and resection [3]. Barnett et al. conducted a systematic review and meta-analysis of LITT (*n* = 79) versus craniotomy (*n* = 1036) of high-grade gliomas near areas of eloquence and found improved extent of treated tissue with reduced complication rates (10% reduction in absolute risk difference, *p* < 0.0001) in the LITT patients [57]. Alattar et al. [58] showed local control of 80–100% of completely ablated lesions and median survivals between 5.8 and 19.8 months in patients receiving LITT for brain metastases after stereotactic radiosurgery. Ivan et al. [59] examined cases of newly diagnosed high grade gliomas treated with LITT and found a median overall survival of 14.2 months and median progression-free survival of 5.1 months. These case series and reviews demonstrate the feasibility of LITT and value of further investigation into its applications.

Laser interstitial thermal therapy leads to a cascade of enzyme induction, protein denaturation, melting of membrane lipids, vessel sclerosis, and coagulative necrosis, driving the intended and predictable thermal ablation [19,60]. Histologic examinations of treated lesions characterize the changes surrounding the laser probe into three primary regions: (1) a central coagulative necrosis; (2) a ring of macrophage-rich granulation tissue; and (3) a peripheral zone of vasogenic edema. Tissue viability increases radially away from the treatment foci as these regions absorb lower levels of the thermal load [16,20,61–63]. A typical setup provides cooling of the laser probe to limit temperature at the tip of the probe to 90 °C to prevent charring, with heat dissipating over distance and establishing a temperature gradient [64]. This temperature gradient is the root of the immunomodulation to be discussed through this review.

### LITT and immunomodulation

**Figure 1.**
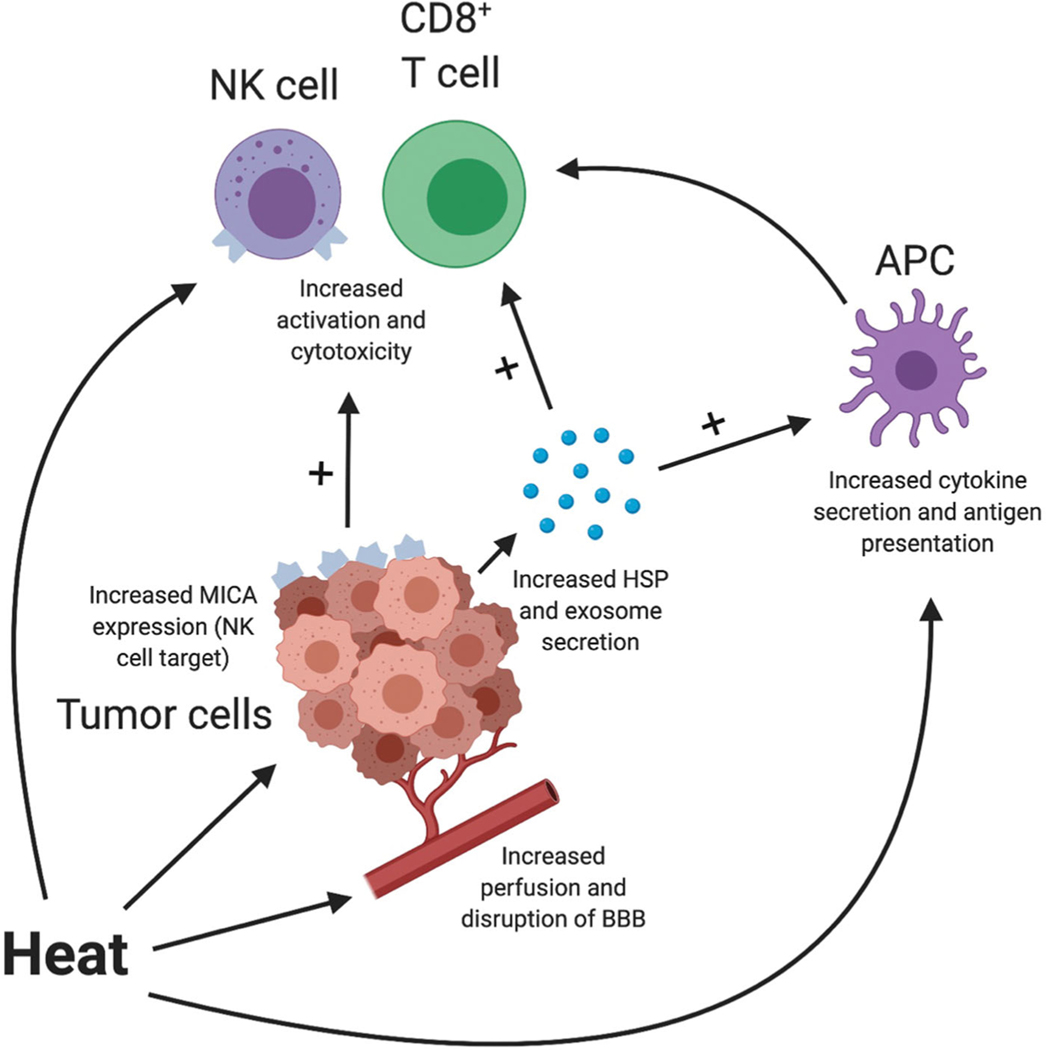
Schematic representing integrated effects of hyperthermia on tumor and immune cells. Created with BioRender.com. APC: antigen-presenting cell; BBB: blood–brain barrier; HSP: heat shock protein; NK cell: natural killer cell.

While cytoreduction *via* thermal ablation within the targeted tumor is the primary tumoricidal effect of LITT, there is evidence that the resultant localized hyperthermia (HT) also modulates and enhances the innate antitumor immune response. Immunologic changes can be broadly categorized into three linked groups of effects: those that impact the tumor cells directly, those that modulate immune cell function and activation, and those that more grossly change the tumor microenvironment (TME). The impacts of localized HT on tumor and immune cells are summarized in Figure 1. Specific changes, to be discussed in more detail below, including the release of tumor antigen-dense exosomes; immune-stimulating heat-shock proteins (HSPs); increased cytokine and chemokine production; enhanced antigen-presenting cell (APC), cytotoxic T cell, and natural killer (NK) cell activity; disruption of the blood–brain barrier (BBB); and vessel dilation with increased perfusion permitting greater immune surveillance [42,65–87]. The collective result is a combination of both immune-stimulating and immunosuppressive effects that modulate the body’s response to treatment and offer the potential to be therapeutically co-opted when HT is combined with IT.

#### Impact on tumor cells

Significant work has been completed to characterize the impact of localized HT on tumor cells themselves. HT causes tumor cell production of heat shock proteins (HSP) and their release into the extracellular environment [71]. HSPs are molecular chaperones that appear in response to heat exposure and have many immunologic functions, such as direct stimulation of NK cells to exert cytotoxic effects and APCs to enhance cytokine release and antigen presentation [67,88–90]. As these HSPs appear in the extracellular space, they are frequently bound to additional intracellular proteins, providing an avenue for cross-presentation of tumor neoantigens on MHC class I or traditional MHC class II presentation [73,91–93]. Udono et al. [81] demonstrated that such cross-presentation, and the associated CD8+ T cell response, results in tumor-specific cytotoxicity. Suzue and Tamura both co-opted the same pathway through administration of HSPs from tumor cells to tumor-naïve mice, demonstrating inhibited progression of primary cancers, reduced metastases, and overall survival benefit [77,94]. Ostberg et al. demonstrated that exposure of *in vitro* tumor target cells to temperatures of 39.5 °C for 6 h resulted in increased expression of MICA, an NK cell target, resulting in enhanced cytotoxicity [75]. Important to drawing inferences from these studies, Nikfarjam et al. [74] showed that laser ablation in a murine model of colorectal liver metastases resulted in greater and more prolonged HSP levels compared to a control of ablated normal liver tissue.

In addition to the upregulation of HSPs, tumor HT also increases the concentration of released tumor exosomes into the TME. Exosomes are small membrane vesicles containing chemokines and concentrated tumor antigens that can be subsequently presented through APCs to stimulate further tumor-specific T cell responses [84,95]. Dai et al. [96] demonstrated that such tumor-derived exosomes can significantly induce dendritic cell (DC) maturation, as well as prime tumor-specific cytotoxic T cells for anti-tumor immunity. Guo et al. found that exosomes from heat-stressed tumor cells stimulated DCs to secrete cytokines converting regulatory T cells (*T*_regs_) into Th_17_ cells and inhibited tumor growth in a murine colon adenocarcinoma model [68]. When considering these findings in the context of applied HT therapies, an important question arises around optimization of the duration and intensity of heat. Notably, many of the results previously described have been identified in the range of normal fever temperatures, generally below 42 °C [65]. There is concern, then, that killing cells prematurely at the higher temperatures that LITT incurs may achieve the cytoreductive goal but fail to generate the desired immunostimulatory response. Understanding the immunologic changes that occur at temperatures upwards of 50 °C will be an important goal moving forward.

#### Direct impact on immune cells

Beyond the tumor cells, HT therapies also have direct impact on immune cells themselves, including APCs, NK cells, T cells, and macrophages. Improved cytotoxicity of CD8+ T cells and NK cells has been demonstrated under HT conditions, as well as increased tumor antigen-specific IFN-γ production [72,75]. Other groups showed that heating DCs to mild fever ranges results in enhanced maturation, antigen uptake, IL-12 production, migration, upregulation of both MHC class I and class II, and T cell stimulation [69,76,80,86]. Van Bruggen et al. [82] showed an analogous activation of macrophages with similar temperature ranges. Isbert et al. [97] demonstrated *in vivo* that LITT compared to resection in rat intrahepatic tumors reduced peritoneal spread and increased expression of CD8 and co-stimulatory molecules. Rats with liver adenocarcinomas treated with laser thermotherapy demonstrated resistance to tumor re-challenge and absence of tumor spread with increased CD8+ T cells compared to resections [98]. Heat treatment by magnetite cationic liposomes in murine glioma subcutaneous flank tumor models resulted in resolution of the treated tumor as well as an untreated tumor on the opposite side, with increased CD8+ and CD4+ T cell tumor activity and infiltration at both locations [99]. Regarding human patients, the immune-modulating impact of LITT therapy has been previously demonstrated in cases on non-CNS tumors. For example, patients with liver metastases of colorectal cancer treated with LITT had increased tumor-specific cytotoxic T cell stimulation with increased cytolytic activity in *in vitro* assays [83].

#### Impact on immune infiltration

The last category to consider in the impact of HT therapies is the broader changes that affect immune cell localization. Heating tumors drives vascular adaptation, as studies in rats showed inducing mild HT to 42.5 °C for 30 min increased the diameter of local arterioles by 35%, blood flow by 50%, and pO_2_ by 50% [100–102]. It also created shifts in IL-6 signaling and subsequently ICAM expression that resulted in increased T cell trafficking into tumors [79]. Localized HT has also been shown to disrupt the BBB, potentially permitting enhanced trafficking of immune cells into the tumor itself [42,103–105]. Leuthardt et al. [104] demonstrated that LITT to recurrent GBMs lead to a disruption of the peritumoral BBB that peaked within 1–2 weeks and resolved after 4–6 weeks. This finding is further supported by evidence that the permeability of BBB is dependent on brain temperature and increases from 38.5 °C before plateauing at 41–42 °C [106]. Morris et al. [42] demonstrated in human epilepsy patients that LITT resulted in disruption of the BBB for up to 8 months after treatment, with one patient developing a delayed optic neuritis. Multiple IT methods including antibodies, targeted toxins, bi-specific T cell engagers (BiTEs), and checkpoint blockade inhibitors have demonstrated issues with tumor infiltration [107–110]. Thus, localized HT may allow increased access to traditional IT modalities to the CNS tumor site, potentially improving their efficacy.

### Combining LITT with IT

Given the range of immunomodulatory impact with localized HT treatments, integration with conventional oncologic treatments was a reasonable next step for the field. The potential of such combinatorial therapies has been demonstrated in preclinical studies finding increased tumor cell lethality for chemotherapy and radiotherapy with HT treatments, and such pairings in human patients have already resulted in improved survival rates [111–113]. With the addition of IT as a treatment modality, the current goal is to use localized HT to flip the tumor environment from an immunosuppressed ‘cold’ state to a ‘hot’ state that is more responsive to checkpoint blockade or adoptive T cell therapies (ACT) [114].

Evidence for the rationale of utilizing HT therapies and IT has been published in pre-clinical studies. Bear et al. demonstrated in a murine metastatic melanoma model that thermo-ablative therapy using gold nano-shells promoted the expression of pro-inflammatory cytokines and chemokines and induced the maturation of DCs within tumor-draining lymph nodes. When combined with the transfer of tumor-specific pmel T-cells, this also prevented primary tumor recurrence as well as inhibiting metastatic tumor growth sites [115]. den Brok et al. showed that adoptive splenocyte transfer from donor mice with tumors heated to ablative temperatures resulted in improved antitumor responses for previously tumor-naïve recipients. Additionally, the same group showed that pairing ablation with an anti-CTLA-4 antibody resulted in protection against tumor re-challenge [94]. Wang et al. showed the combination of local photothermal ablation using single-walled carbon nanotubes with anti-CTLA-4 therapy prevented the development of distant tumor metastases in a murine lung cancer model, along with prolonged animal survival [116]. Han et al. demonstrated that thermal ablation of murine flank colorectal tumors followed by administration of toll-like-receptor agonists and anti-CTLA-4 therapies resulted in destruction of tumors at distant sites with a significant increases in their CD8+ T cell to T_reg_ ratio, as well as long-term resistance to tumor re-challenge [117]. Luo et al. showed that tumor ablation followed by PD-1 blockade in murine breast and lung cancer models resulted in primary tumor resolution as well as a systemic immune response that suppressed metastatic lesions and re-challenge [118]. Similar results have been demonstrated in murine models of neuroblastoma, colon cancer, and breast cancer [119,120]. Applications of combined HT and IT in preclinical malignant glioma models are currently limited in the literature. One study utilized a murine flank GBM model to test the pair. This group developed a novel method to generate gold nanoparticles that selectively accumulated in tumors and amplified the effect of light-based photothermal ablation in a treatment mechanism analogous to LITT. When paired with anti-PD-L1 antibodies, the combined treatment group demonstrated reduced tumor growth and improved survival relative to controls and each therapy in isolation, as well as lasting immunologic memory that rejected tumor re-challenge [121]. These results support further investigation into the integration of thermal therapy and IT, and the potentially synergistic effect of the two on local and metastatic lesions as well as long-term antitumor immunologic memory.

Currently, there are few published data on pairing HT with IT in human patients (Table 1). In one small case series of just two patients, Paiva et al. [122] showed extended survival with renal cell carcinoma metastases to the head and neck treated with laser-induced thermal therapy and IL-2. In a pilot study of patients with ovarian, pancreatic, gastric, colorectal, cervical, or endometrial cancer, 33 patients received local HT therapy plus ACT alone, or with either salvage chemotherapy or anti-PD-1 antibody. Seven out of 10 patients with local HT plus ACT, and 6 out of 11 patients treated additionally with anti-PD-1, had disease control. Overall, the study had an objective response rate of 30% with significantly increased cytokine markers among the clinical responders and a favorable toxicity profile among all groups [123].

Beyond these results, there are several ongoing or upcoming phase I/II trials targeting CNS pathologies with LITT/HT in addition to IT regimens (Table 2). The available information indicates that these trials will investigate the pairing with checkpoint blockade therapy targeting the PD-1/PD-L1 axis with either pembrolizumab or avelumab in recurrent GBM as well as in CNS metastases from melanoma, non-small cell lung carcinoma (NSCLC), and renal cell carcinoma. A group out of Mount Sinai (NCT03341806) is currently enrolling 30 patients with recurrent GBM to receive LITT followed by IV avelumab every 2 weeks, compared to avelumab alone, with measured outcomes of dose limiting toxicity, objective response rate, progression-free survival, and overall response rate [124]. An upcoming trial at the University of Florida (NCT04187872) will entail LITT followed by pembrolizumab every 3 weeks until recurrence for up to 2 years in patients with brain metastases from melanoma, non-small cell lung carcinoma, or renal carcinoma that have recurred after stereotactic radiosurgery [125]. Lastly, a trial at Case Comprehensive Cancer Center (NCT03277638) is currently recruiting patients with recurrent GBM for LITT with administration of pembrolizumab 7 days before, 14 days after, or 35 days after treatment [126]. Additional trials exploring the combination of HT and IT in extracranial disease are summarized in Table 3.

## Conclusion

Ultimately, the integration of therapeutic modalities and expansion of our toolset in oncologic disease are important steps forward. The recent rise of IT has substantially shifted the context of these investigations. However, its success in tumors of the CNS remains limited and therefore combination with treatments that may improve access and effectiveness are a valuable avenue of research. The immunomodulatory impacts of localized HT have been known and investigated for some time, with demonstrated effects at multiple levels of the antitumor immune response. Given its immunostimulatory potential, pairing localized HT with the continually evolving IT strategies is a promising path with supportive preclinical data. As both HT and IT are pushed forward with ongoing independent research, the trials above represent the current next steps as we work toward characterization and optimization of their combination.

## Figures and Tables

**Table 1.** Published hyperthermia (HT) with immunotherapy (IT) studies in human patients.

Author	HT method	IT Agent	Population	Outcome	Complications
Paiva et al.	LITT	IL-2 infusion in the week after LITT	Two adult patients with renal cell carcinoma metastases to the head and neck	Patient 1 passed 2 years later from metastases to the lungs Patient 2 passed 20 months after treatment	None
Qiao et al.	RFA	10 received ACT, 11 received ACT and pembrolizumab, 12 received ACT and CT	33 patients with ovarian, pancreatic, gastric, colorectal, cervical, or endometrial cancer	30% objective response rate, 66.7% disease control rate with 9.1% complete responses and 21.2% partial responses	Blistering (3/33), subcutaneous fat induration (4/33), local hear-related pain (3/33), vomiting (1/33), and sinus tachycardia (1/33)

ACT: adoptive T cell therapy; CT: chemotherapy; LITT: laser interstitial thermal therapy; RFA: radiofrequency ablation.

**Table 2.** Intracranial hyperthermia (HT) with immunotherapy (IT) clinical trials.

Clinical trial indicator	HT method	IT agent	Population	Phase	Status
NCT03341806	LITT	Avelumab	Adults with recurrent GBM	I	Recruiting
NCT04187872	LITT	Pembrolizumab	Adults with recurrent brain metastases from primary melanoma, non-small cell lung cancer, or renal cell carcinoma	I	Not yet recruiting
NCT03277638	LITT	Pembrolizumab	Adults with recurrent GBM	I/II	Recruiting
NCT03939975	RFA	Pembrolizumab	Adults with advanced refractory hepatocellular carcinoma	II	Completed
NCT03993678	LITT	N-dihydro-galactochitosan	Adults with advanced or recurrent solid tumors	I	Not yet recruiting

GBM: glioblastoma; LITT: laser interstitial thermal therapy; RFA: radiofrequency ablation.

**Table 3.** Extracranial hyperthermia (HT) with immunotherapy (IT) clinical trials.

Clinical trial indicator	HT method	IT agent	Population	Phase	Status
NCT03757858	RFA	ACT, ACT plus pembrolizumab, ACT plus standard CT, or standard CT	Adults with abdominal or pelvic malignancies or metastases	I/II	Recruiting
NCT03393858	RFA	ACT and pembrolizumab	Adults with advanced malignant mesothelioma	I/II	Recruiting

ACT: adoptive T cell therapy; CT: chemotherapy; RFA: radiofrequency ablation.
